# A RIPK2-associated macrophage program links mycobacterial sensing to osteoclastogenesis in bone tuberculosis

**DOI:** 10.3389/fcimb.2026.1970405

**Published:** 2026-10-08

**Authors:** Yuyang Zhang, Ziyang Zhang, Dongyang Zhang, Jiulin Tan, Zhaoxin Ma, Qingyang Xiao, Jie Zhang, Qixiu Yu, Zehua Zhang, Yong Tang, Ce Dou, Fei Luo

**Affiliations:** 1 Department of Orthopedics, Southwest Hospital, Army Medical University, Chongqing, China; 2 School of Medical and Information Engineering, Gannan Medical University, Ganzhou, Jiangxi, China; 3 Department of Orthopaedics, 72nd Group Army Hospital, Huzhou Normal University, Huzhou, Zhejiang, China

**Keywords:** bone tuberculosis, GSK583, osteoclastogenesis, RIPK2, super-enhancer

## Abstract

**Introduction:**

In bone tuberculosis, Mycobacterium tuberculosis (Mtb) infection triggers inflammatory bone destruction, but the macrophage programs that convert Mtb signals into osteoclastogenesis remain poorly defined.

**Methods:**

We generated single-cell RNA-seq data from human spinal tuberculosis lesions and integrated them with public H3K27ac ChIP-seq, RNA-seq, Hi-C, and motif data. Key findings were validated in primary macrophages stimulated with Mtb lysate.

**Results:**

We identified 64 Mtb-induced super-enhancers, and integrated analysis prioritized RIPK2 as a candidate. Lesion-associated myeloid populations showed enhanced inflammatory and osteoclast-priming signatures, followed a mononuclear phagocyte-to-osteoclast trajectory, and exhibited a linear correlation between RIPK2 expression and osteoclast score. In mice, RIPK2 localized to CTSK-positive bone interfaces. *In vitro*, Mtb stimulation induced *Il1b, Nfkbiz*, and *Tnfrsf11a* expression, IL-1β secretion, and surface RANK upregulation; these responses were suppressed by the RIPK2 inhibitor GSK583.

**Discussion:**

These findings link RIPK2 to both macrophage inflammatory signaling and osteoclastogenesis in bone tuberculosis, suggesting that RIPK2 may be a target for limiting infection-driven bone destruction.

## 1 Introduction

Bone tuberculosis is a destructive form of osteoarticular tuberculosis characterized by persistent inflammation, progressive bone erosion, deformity, and long-term functional impairment (Ma et al., 2022). Dysregulated osteoclastogenesis and disruption of the RANKL–osteoprotegerin balance are central features of this pathological bone loss, and experimental models of mycobacterial bone infection consistently demonstrate increased accumulation and activity of tartrate-resistant acid phosphatase-positive osteoclasts (Liu et al., 2025; Jia et al., 2026). Although antimicrobial therapy is essential for controlling infection, it does not directly address the host inflammatory processes that promote continued osteoclast activation and local bone resorption. A key unresolved question is therefore which macrophage-associated inflammatory pathways connect mycobacterial sensing to osteoclastogenic bone destruction. Identifying pharmacologically tractable regulators of this interface may provide a rationale for adjunctive host-directed strategies aimed at preserving bone integrity during tuberculosis treatment.

Macrophages are major responders to *Mycobacterium tuberculosis* (MTb) and, together with osteoclasts, arise from the monocyte–macrophage lineage (Cai et al., 2024). Exposure to mycobacterial products activates pattern-recognition receptor, cytokine, interferon, and metabolic pathways that reshape the local inflammatory microenvironment and may influence osteoclast differentiation directly or through interactions with stromal and osteolineage cells (Russell et al., 2025). Among these pathways, nucleotide-binding oligomerization domain-containing protein 2 (NOD2) detects peptidoglycan-derived ligands and recruits receptor-interacting serine/threonine-protein kinase 2 (RIPK2) to activate NF-κB and MAPK signaling (Pandey et al., 2009). NOD2–RIPK2 signaling has also been implicated in osteoclast regulation, though its effects vary with the inflammatory context and experimental setting (Li et al., 2025). Whether a pharmacologically responsive RIPK2-associated macrophage program contributes to osteoclastogenic bone injury in skeletal tuberculosis remains unclear.

Super-enhancers (SEs) are clusters of regulatory elements marked by high levels of histone H3 lysine 27 acetylation (H3K27ac) and are frequently associated with genes that control cell identity and disease-relevant transcriptional states (Pott and Lieb, 2015). Infection-induced remodeling of the macrophage super-enhancer landscape may therefore reveal regulatory nodes that are not apparent from transcriptomic analysis alone (Jia et al., 2019). However, super-enhancer classification is highly sensitive to rank thresholds, and simple subtraction of condition-specific enhancer lists can exaggerate apparent selectivity for regions located near the calling boundary (Wang et al., 2022). To reduce this bias, we defined Mtb-responsive super-enhancers using a shared enhancer universe, rank-ordered state shifts, and quantitative H3K27ac signal gain, and then integrated these regions with matched transcriptional and chromatin-contact evidence.

In this study, we integrated multi-omics profiling to characterize Mtb-induced macrophage inflammatory network remodeling and identified RIPK2 as a pharmacologically targetable node and assessed whether this RIPK2-associated inflammatory axis primes RANK-driven osteoclast formation in bone tuberculosis.

## 2 Methods

### 2.1 Ethics approval and consent to participate

This study was approved by the faculty of Army Medical University (Third Military Medical University, KY2024198, AMUWEC20223775) and carried out in accordance with the requirements of Army Medical University (Third Military Medical University).

### 2.2 Public datasets acquisition

We reanalyzed public mouse BMDM datasets, including H3K27ac ChIP-seq (GSE98598) and matched RNA-seq (GSE98599) from Mtb-infected versus controls, together with a public BMDM Hi-C dataset (GSE115524).

### 2.3 ChIP-seq preprocessing and common enhancer universe

H3K27ac FASTQ files were aligned to the mm10 mouse genome with Bowtie2 v2.3.5.1, and H3K27ac peaks were called against matched input with MACS2 v2.2.6 in broad-peak mode. Replicate-level broadPeak outputs were merged with BEDTools to build a shared enhancer universe across conditions, making cross-condition rank and signal comparisons comparable and avoiding interpretation of peak-calling differences as selective SE biology.

### 2.4 Super-enhancer identification

Promoter-proximal regions within ±2 kb of annotated transcription start sites were removed before SE calling. Replicate-level ROSE-style ranking was then performed, and enhancer rank and H3K27ac signal were averaged across replicates. ROSE was run with version 1.3.2, a stitching distance of 12.5 kb (-s 12500) and a TSS exclusion window of ±2.5 kb (-t 2500); the ROSE TSS exclusion is separate from the upstream ±2 kb promoter filter (Huang et al., 2018). A uniform mean-rank cutoff of 1382 was applied across all conditions. Mtb-induced SEs were not defined by overlap subtraction. The high-confidence set required Mtb final-SE status, absence from PBS final SEs and Mtb/PBS H3K27ac fold change ≥1.5. The stringent set required Mtb/PBS log2FC > 1.

### 2.5 SE-target assignment and transcriptomic integration

SE-linked genes were assigned by refGene overlap, nearest-TSS and proximal 100-kb rules. These are proximity-based putative targets, not confirmed enhancer-promoter assignments. Targets were merged with GSE98599 differential expression and prioritized by SE signal gain, RNA log2FC, FDR and contact context.

### 2.6 Functional enrichment and motif analysis

Gene Ontology and KEGG enrichment used clusterProfiler v4.4.4 (Wu et al., 2021). Motif analysis used HOMER findMotifsGenome.pl v5.1 against mm10, with constituent H3K27ac peaks inside Mtb-induced SEs as functional-region approximations and matched non-selective H3K27ac peaks as background (-size given -mask -len 8, 10, 12 -p 16). Motif-linked transcription-factor programs were interpreted as hypotheses about pathway activity, not evidence of direct occupancy.

### 2.7 Chromatin-contact context

Public mouse BMDM Hi-C data were obtained from GSM3181429 within GEO series GSE115524, and the processed GSM3181429_BMDM_HiC_B1.hic file was used for contact-matrix extraction (Mumbach et al., 2019). For each representative locus, a genomic window spanning the SE interval and the transcription start site (TSS) of the assigned candidate gene was defined. KR-normalized observed contact matrices were extracted from the.hic file at 10-kb resolution using strawr (Durand et al., 2016). Sparse bin-pair contacts were converted into symmetric dense matrices for visualisation. Promoter-centred virtual 4C profiles were generated from these observed contact matrices by selecting the 10-kb bin closest to the target-gene TSS as the viewpoint and extracting its contact vector across the corresponding locus (Yardimci and Noble, 2017). The bin closest to the SE centre was highlighted as the SE-centre bin.

For the distance-normalized contact analysis, KR-normalized observed/expected (O/E) values were separately extracted from the same public.hic file at 10-kb resolution for the bins containing each SE centre and target-gene TSS. Candidate pairs with an O/E value ≥1.5 and an SE-centre-to-TSS distance ≥20 kb were operationally classified as showing O/E-enriched local contact. Pairs separated by <20 kb were considered promoter-proximal contact neighbourhoods and were not counted as resolved enriched SE–promoter contacts. This operational threshold was used for descriptive prioritisation and was not interpreted as formal loop-calling significance, direct enhancer regulation or an Mtb-specific differential chromatin interaction. Local Hi-C maps were visualized using the R package gghic (Jiang et al., 2025).

### 2.8 Human bone tuberculosis scRNA-seq analysis

Single-cell suspensions from spinal tuberculosis tissues (n = 6) were processed by Juyuan Gene Tech using the Chromium Next GEM Single Cell 3′ v3.1 platform (10x Genomics), targeting approximately 10, 000 cells per sample. Libraries were sequenced on an Illumina NovaSeq 6000 at approximately 50, 000 reads per cell, and gene–barcode count matrices were generated using Cell Ranger v4.0.0. Non-tuberculosis scRNA-seq datasets comprising 11 samples were obtained from GEO accessions GSE242414 and GSE253355.

All datasets were analyzed in Seurat v4.3.0 using standard quality-control (nCount_RNA > 3000 & nFeature_RNA>200), normalisation, dimensional-reduction, clustering and UMAP workflows. Samples were integrated with Harmony v0.1.1, and cell types were annotated using canonical lineage markers. Osteoclast-differentiation and inflammatory module scores were calculated from predefined gene signatures and visualized on UMAP. Gene-set enrichment analysis of combined myeloid cells was performed using GSEApy. Expression was summarised at the donor level for pseudobulk and correlation analyses. Cell-cell communication and pseudo-time analysis were used R package CellChat (v2.2.0.9001) and monocle2 (v2.22.0) according to the tutorial respectively. Cells were ordered using genes that differed across the selected lineage states, and the root state was assigned to the mononuclear-phagocyte-enriched branch based on canonical lineage-marker expression. Osteoclast-differentiation and inflammatory transcriptional programs were quantified at the single-cell level using UCell. The osteoclast-differentiation signature comprised TNFRSF11A, NFATC1, ACP5, CTSK, DCSTAMP, OCSTAMP, ATP6V0D2, OSCAR, CLCN7, TCIRG1, CALCR, and SIGLEC15. The inflammatory signature comprised IL1B, TNF, NFKBIZ, NFKBIA, PTGS2, CXCL8, CXCL2, CCL2, CCL3, CCL4, NLRP3, SOCS3, and ICAM1.

### 2.9 Mouse bone tuberculosis model, microCT and histology

Animal experiments were approved by the institutional animal ethics committee and performed under appropriate biosafety containment. Control mice received normal saline injections. For the virulent Mtb model, eight- to ten-week-old C57BL/6 male mice received intratibial injection of 2 μL of *Mycobacterium tuberculosis* suspension at 1 × 10^8^ CFU/mL, whereas control animals were administered an equivalent volume of sterile saline (n=4, per group). After 4 weeks, the mice were sacrificed. Following overnight fixation in 4% paraformaldehyde, murine femora, tibiae and vertebrae were scanned using a SkyScan 1272 microCT system (Bruker, Kontich, Belgium) as previously described (Sun et al., 2022). Scans were acquired at 7-μm isotropic resolution, 60 kV, 166 μA and an exposure time of 1700 ms per projection. Then bone specimens were decalcified in EDTA, embedded, sectioned, and subjected to hematoxylin and eosin (H&E) staining to assess inflammatory lesion architecture and tartrate-resistant acid phosphatase (TRAP) staining to evaluate osteoclast accumulation. For immunofluorescence staining, bone sections were blocked with 5% bovine serum albumin in phosphate-buffered saline (PBS) and incubated overnight at 4 °C with primary antibodies against RIPK2 (Proteintech; 1:100) and the osteoclast-lineage marker cathepsin K (CTSK; Proteintech; 1:100). After washing with PBS, the sections were incubated with appropriate fluorophore-conjugated secondary antibodies for 1 h at 37 °C. Nuclei were counterstained with DAPI, and fluorescence images were acquired using a Zeiss LSM 880 confocal laser-scanning microscope.

### 2.10 Primary BMDM culture and Mtb lysate stimulation

Primary bone marrow-derived macrophages (BMDMs) were generated as previously described (Toda et al., 2021) and cultured in α-Minimum Essential Medium (α-MEM; Gibco) supplemented with 10% fetal bovine serum and 1% penicillin–streptomycin. Cells were assigned to four treatment groups: DMSO vehicle control, 1 μM GSK583 (MedChemExpress, HY-100339) as previously described (Steinle et al., 2021), 10 μg/mL heat-killed Mtb lysate (Shanghai Gene-Optimal Science & Technology Co., Ltd), or GSK583 plus Mtb lysate. CCK-8 reagent was added according to the manufacturer’s instructions, and absorbance was measured at 450 nm. Cell viability was expressed relative to the vehicle-treated control. GSK583 and Mtb lysate were added simultaneously, and cells were incubated for 24 h. The final DMSO concentration was matched across all groups. After treatment, total RNA was isolated using RNA Extraction Kits (TianGen, China) and reverse-transcribed using Thermo Scientific RevertAid RT Kit. Quantitative real-time PCR was performed using TargetMol SYBR Green qPCR Master Mix to determine the expression of *Il1b, Nfkbiz* and *Tnfrsf11a*. Expression was normalized to *Actb*, and relative expression was calculated using the 2^−ΔΔCt^ method. The primer sequences used in this study are listed in **Supplementary Table 2**.

### 2.11 IL-1β ELISA and immunoblotting

Culture supernatants were collected after 24 h and clarified by centrifugation. IL-1β concentrations were measured using a mouse IL-1β ELISA kit (Jianglai Biotechnology, Shanghai, China) according to the manufacturer’s instructions. Absorbance was measured at 450 nm, and cytokine concentrations were calculated from the standard curve and expressed as pg/mL. For immunoblotting, BMDMs were washed with ice-cold PBS and lysed in RIPA buffer supplemented with protease/phosphatase inhibitors. Protein concentrations were determined using a BCA assay. Equal amounts of protein were separated by SDS–PAGE and transferred to PVDF membranes. Membranes were blocked with 5% non-fat milk and incubated overnight at 4 °C with antibodies against RIPK2 (Proteintech, 15366-1-AP), TNF-a (Proteintech, 17590-1-AP) and β-actin (Proteintech, 66009-1-Ig). After incubation with the appropriate horseradish peroxidase-conjugated secondary antibodies, protein bands were detected using enhanced chemiluminescence.

### 2.12 Flow-cytometric analysis of cell-surface RANK

After 24 h of treatment, BMDMs were gently detached, washed with flow-cytometry staining buffer and incubated with APC-conjugated anti-mouse CD265/RANK antibody (clone R12-31; BioLegend) for 30 min at 4 °C in the dark. After washing, samples were acquired using a BD LSRFortessa flow cytometer and analyzed using FlowJo software. RANK expression was quantified as the median fluorescence intensity within the singlet BMDM population.

### 2.13 Osteoclast differentiation and TRAP staining

Primary BMDMs were cultured in 24-well plates in the presence of 20 ng/mL M-CSF and 75 ng/mL RANKL. Cells were assigned to a 2 × 2 factorial treatment design according to the presence or absence of heat-killed Mtb lysate (10 μg/mL) and GSK583 (1 μM), yielding four groups: vehicle control, GSK583 alone, Mtb lysate alone, and GSK583 plus Mtb lysate. On day 5, cells were fixed and stained using a TRAP staining kit (GENMED Scientifics Inc., Shanghai, China) according to the manufacturer’s instructions. Whole-well and microscopic images were acquired under identical settings. A TRAP-positive multinucleated osteoclast was defined as a TRAP-positive cell containing at least three nuclei. TRAP-positive multinucleated osteoclasts were counted across the entire well. Three independent biological experiments were performed, and each biological replicate was represented by one independently cultured well per treatment condition. Where technical replicate wells were included, their values were averaged before statistical analysis.

### 2.14 Statistical analysis

Data are mean ± SEM unless stated otherwise. Two groups were compared by unpaired two-tailed Student t test or Mann-Whitney U test according to distribution; multiple groups by one- or two-way ANOVA followed by Tukey’s multiple-comparison test in GraphPad Prism 9. P<0.05 was considered significant. Significance levels are indicated as *p < 0.05, **p < 0.01, and ***p < 0.001, ****P < 0.0001.

## 3 Results

### 3.1 Identification of Mtb-induced macrophage SEs landscapes

To minimize threshold bias in super-enhancers (SEs) calling, we built a common enhancer pool from replicate-level H3K27ac peaks, excluded promoter-proximal regions, ran ROSE on each replicate separately, and applied the same mean-rank cutoff (1, 382) across all conditions (**Figure 1A**). The resulting rank distributions and SE counts were similar: 1, 372 in PBS controls, 1, 369 in Mtb-stimulated, 1, 378 in Bacillus Calmette-Guérin (BCG)-treated, and 1, 358 in the BCG+Mtb group (**Figures 1B, C**). UpSet plots showed that 1, 086 SEs were shared by all four conditions, with smaller condition-specific or partially shared subsets (**Figure 1D**).

**Figure 1:**
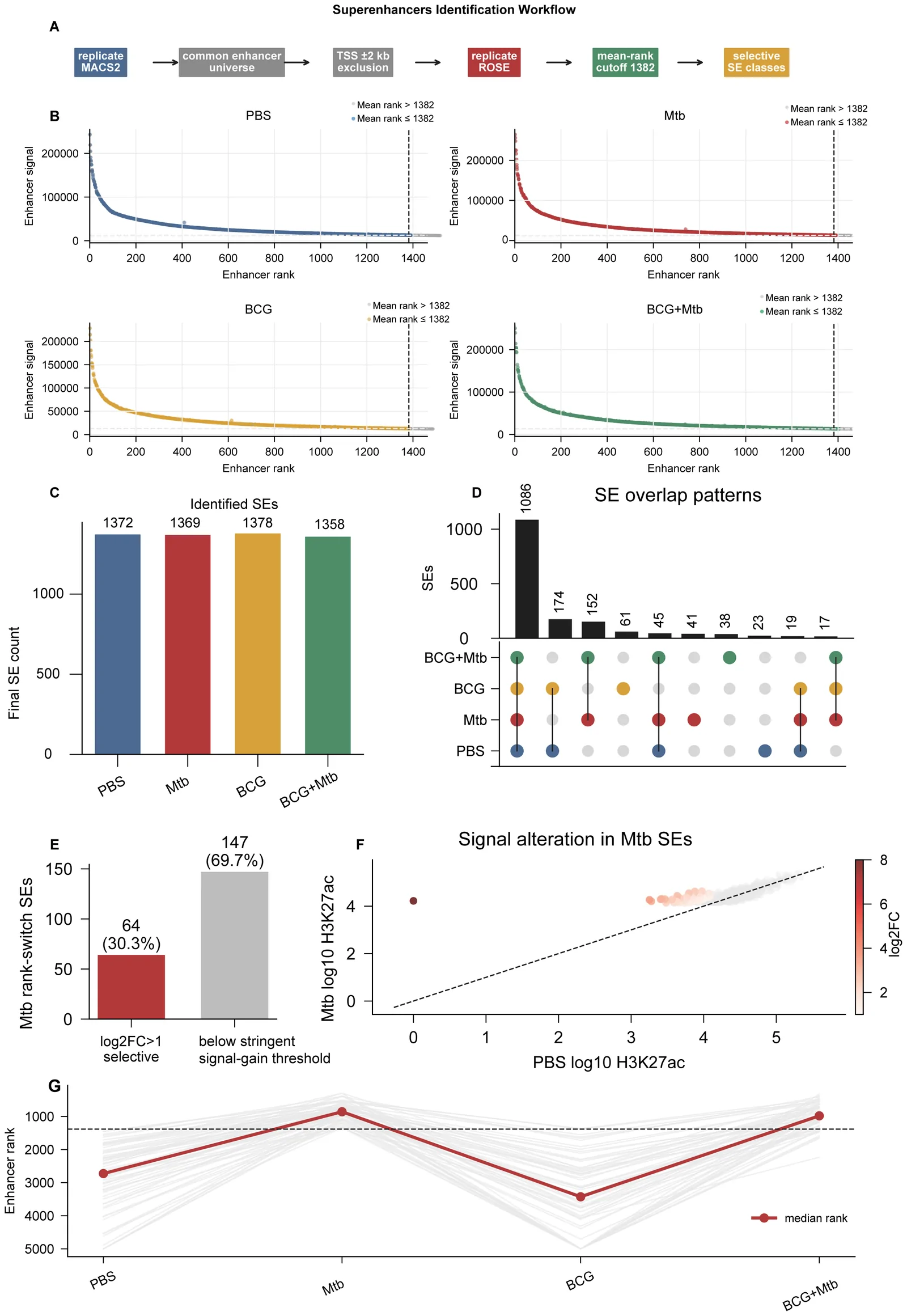
Identification of Mtb-induced macrophage SE landscapes. **(A)** Overview of the replicate-level SE identification workflow. **(B)** ROSE enhancer-rank curves for PBS-, Mtb-, BCG- and BCG+Mtb-treated BMDMs. **(C)** Numbers of final SEs identified in PBS (1, 372), Mtb (1, 369), BCG (1, 378) and BCG+Mtb (1, 358). **(D)** UpSet plot showing shared and condition-associated SEs, including 1, 086 SEs common to all four conditions. **(E)** Signal-gain classification of 211 Mtb-specific rank-switch SEs that reached final-SE status in Mtb. **(F)** Comparison of H3K27ac signal in PBS and Mtb at the 64 stringent SEs. **(G)** Enhancer-rank trajectories of the 64 stringent Mtb-induced super-enhancers across treatment conditions.

To further narrow down, we first identified 211 SEs that entered the final-SE range only in Mtb-stimulated BMDMs. Of these, 64 showed a signal greater than twofold H3K27ac increase (log_2_FC >1), and we took these as our high-confidence Mtb-induced SEs (**Figure 1E**, **Supplementary Table S1**). They were consistently enriched in Mtb samples, falling above the equality line (**Figure 1F**), and their median rank moved into the SE territory upon infection (**Figure 1G**). We thus obtained a conservative set of 64 SEs responsive to Mtb, defined by both rank transition and quantitative signal gain.

### 3.2 Motif and transcriptomic integration identifies inflammatory regulatory programs associated with Mtb-induced SEs

We investigated which transcription factors might be driving these 64 Mtb-induced SEs. Motif scanning returned several families, including NF-κB, nuclear receptor, NFAT, AP-1/CREB, IRF/BATF, ETS/PU.1, and STAT, and all of which have known associated to inflammatory or myeloid regulation (**Figure 2A**). The matched RNA-seq data confirmed that representative members of each family were upregulated in infected BMDMs (**Figure 2B**). Overall, these SEs sit within a regulatory landscape shaped by NF-κB, AP-1, interferon, and myeloid-lineage signals.

**Figure 2:**
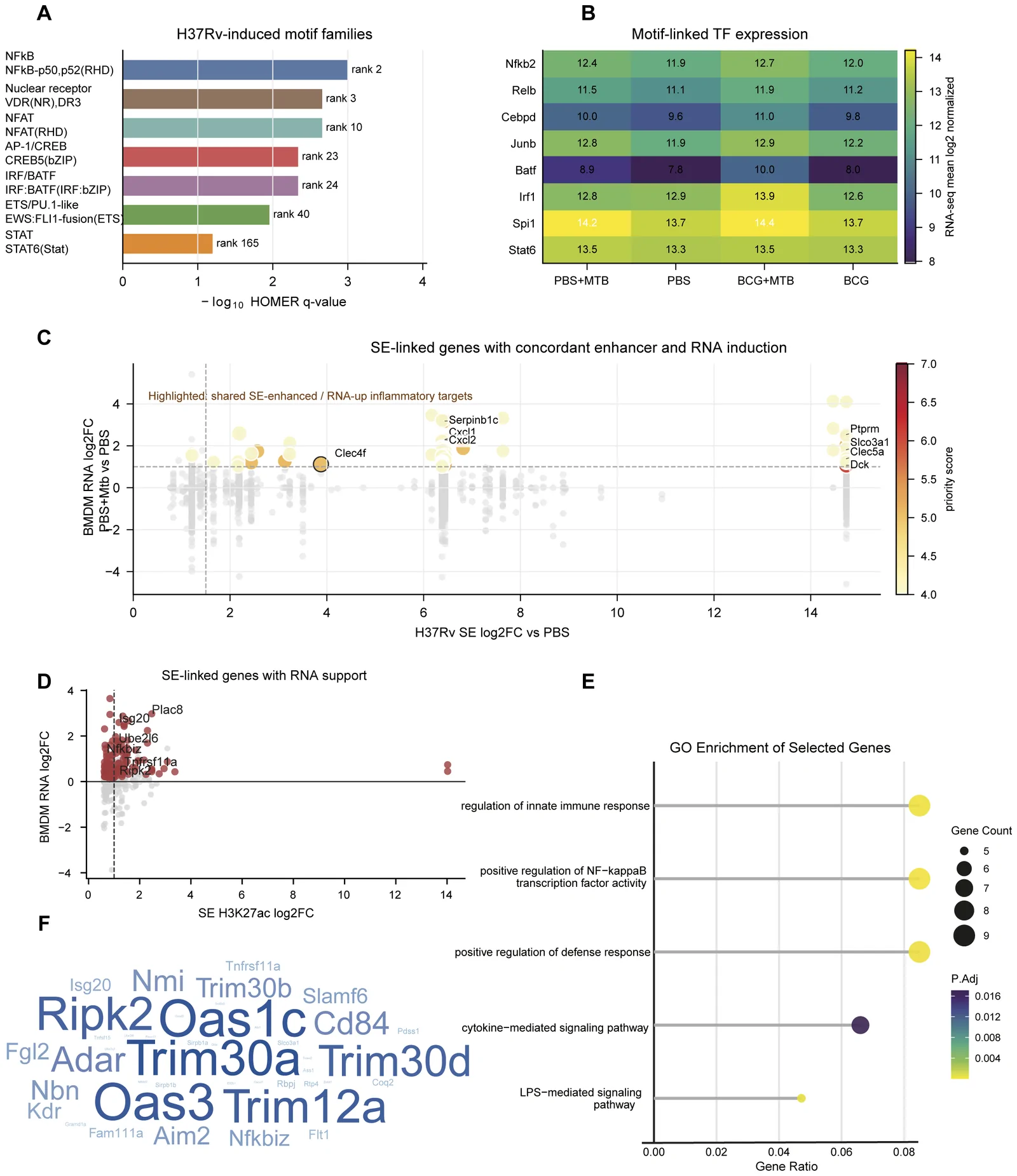
Motif and Transcriptomic Integration Identifies Inflammatory Regulatory Programs Associated with Mtb-Induced Super-Enhancers. **(A)** Representative transcription-factor motif families enriched within constituent H3K27ac peaks of the 64 stringent Mtb-induced super-enhancers. **(B)** Heatmap shows mean expression of selected motif-linked transcription factors in PBS-, Mtb-, BCG-, and BCG+Mtb-treated BMDMs based on matched RNA-sequencing data. **(C)** Integration of Mtb-associated super-enhancer H3K27ac gain with matched transcriptional responses in BMDMs. **(D)** Prioritization of super-enhancer-linked genes with transcriptional support. **(E)** Gene Ontology enrichment analysis of the selected concordantly induced super-enhancer-linked genes. **(F)** Word cloud summarizing genes represented across the enriched inflammatory and host-defense Gene Ontology categories.

We then checked genes whose SE activation aligned with transcriptional upregulation upon infection. This analysis identified a subset of genes showing concordant enhancer activation and RNA upregulation, including inflammatory and host-response genes such as *Cxcl1*, *Cxcl2*, *Serpinb1c*, *Clec4f*, *Clec5a*, and *Dck* (**Figure 2C**). We further singled out six candidates: *Ripk2*, *Nfkbiz*, *Tnfrsf11a*, *Ube2l6*, *Isg20*, and *Plac8* (**Figure 2D**). These genes were enriched for innate immune and defense responses, including positive regulation of NF-κB activity, cytokine-mediated signaling, and lipopolysaccharide-associated pathways (**Figure 2E**). The genes contributing to these enriched functions were summarized to illustrate the inflammatory and host-defense composition of the prioritized program (**Figure 2F**). Together, these analyses linked Mtb-induced SEs remodeling to coordinated inflammatory transcriptional responses and nominated *Ripk2* and related candidates for subsequent locus-level chromatin-contact assessment.

### 3.3 Chromatin-contact analysis provides locus-level context for prioritized super-enhancer-linked candidates

We analyzed public BMDM Hi-C maps using local 10-kb KR-normalized contact matrices, overlaid with H3K27ac tracks. At the *Ripk2*, *Tnfrsf11a*, and *Nfkbiz* loci, the Mtb-induced SE intervals fell within contact domains that also contained the corresponding target promoters (**Figure 3A**). Promoter-centered virtual 4C profiles from the same matrices showed contact signals reaching from the promoter viewpoints toward or across those SE intervals (**Figure 3B**). These patterns gave us some descriptive context for the proximity-based assignments, but we did not treat them as evidence of direct regulatory activity.

**Figure 3:**
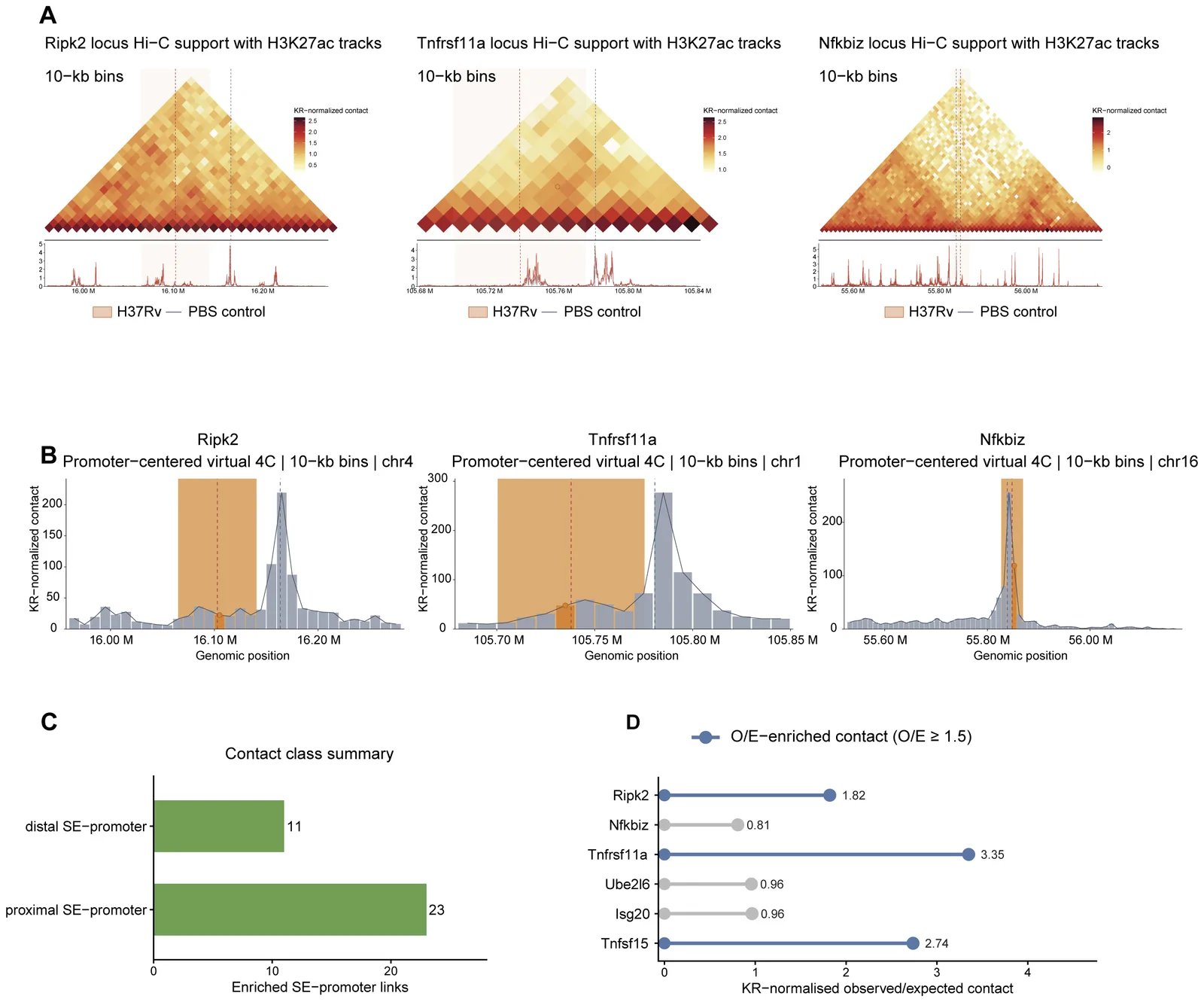
Chromatin-Contact Analysis Provides Locus-Level Context for Prioritized Super-Enhancer-Linked Candidates. **(A)** Local 10-kb KR-normalized observed Hi-C contact maps at the Ripk2, Tnfrsf11a, and Nfkbiz loci, shown together with Mtb and PBS H3K27ac signal tracks. **(B)** Promoter-centered virtual 4C profiles generated from the corresponding Hi-C matrices using the 10-kb bin nearest the target-gene transcription start site as the viewpoint. **(C)** Distance classification of candidate super-enhancer–promoter pairs showing observed/expected-enriched local contact signal. Among the 34 pairs meeting the predefined enrichment criterion, 23 were classified as proximal contacts spanning 20–100 kb and 11 as distal contacts spanning more than 100 kb. **(D)** KR-normalized observed/expected contact values for selected candidate super-enhancer–promoter pairs. The dashed line indicates the predefined enrichment threshold of observed/expected ≥1.5.

We then applied a distance-normalized observed/expected contact criterion to assess the broader candidate pool. Among 34 pairs that showed enriched local contact signal, 23 were proximal (20–100 kb) and 11 were distal (>100 kb) (**Figure 3C**). For Ripk2, Tnfrsf11a, and Tnfsf15, the observed/expected scores came out at 1.82, 3.35, and 2.74, all above our 1.5 enrichment threshold (**Figure 3D**). We took this as supportive context for prioritization, while keeping in mind that these data do not demonstrate direct enhancer–promoter causality or Mtb-specific looping.

### 3.4 Human single-cell analysis reveals coordinated inflammatory and osteoclastogenic remodeling in bone tuberculosis

We used single-cell RNA-seq from spinal tuberculosis (TB) tissues to examine inflammatory bone remodeling in a human disease, with non-infected samples as reference. UMAP resolved nine major cell types, including T cells, B-lineage cells, neutrophils, mononuclear phagocytes, dendritic cells, osteolineage/stromal cells, osteoclasts, endothelial cells, and erythroid cells (**Figure 4A**). Canonical marker expression supported these assignments (**Figure 4C**). TB samples showed greater osteoclast abundance and a redistributed myeloid compartment compared with the reference condition (**Figure 4B**). Osteoclast-differentiation scores were highest in osteoclasts and adjacent myeloid clusters, while inflammatory scores were more broadly distributed across mononuclear phagocytes, neutrophils, and osteoclast-associated cells (**Figure 4D**). In TB samples, both signatures occupied larger UMAP regions and shifted toward higher per-cell scores (**Figure 4E**), pointing to an expanded osteoclast-lineage population with enhanced inflammatory priming in the myeloid compartment.

**Figure 4:**
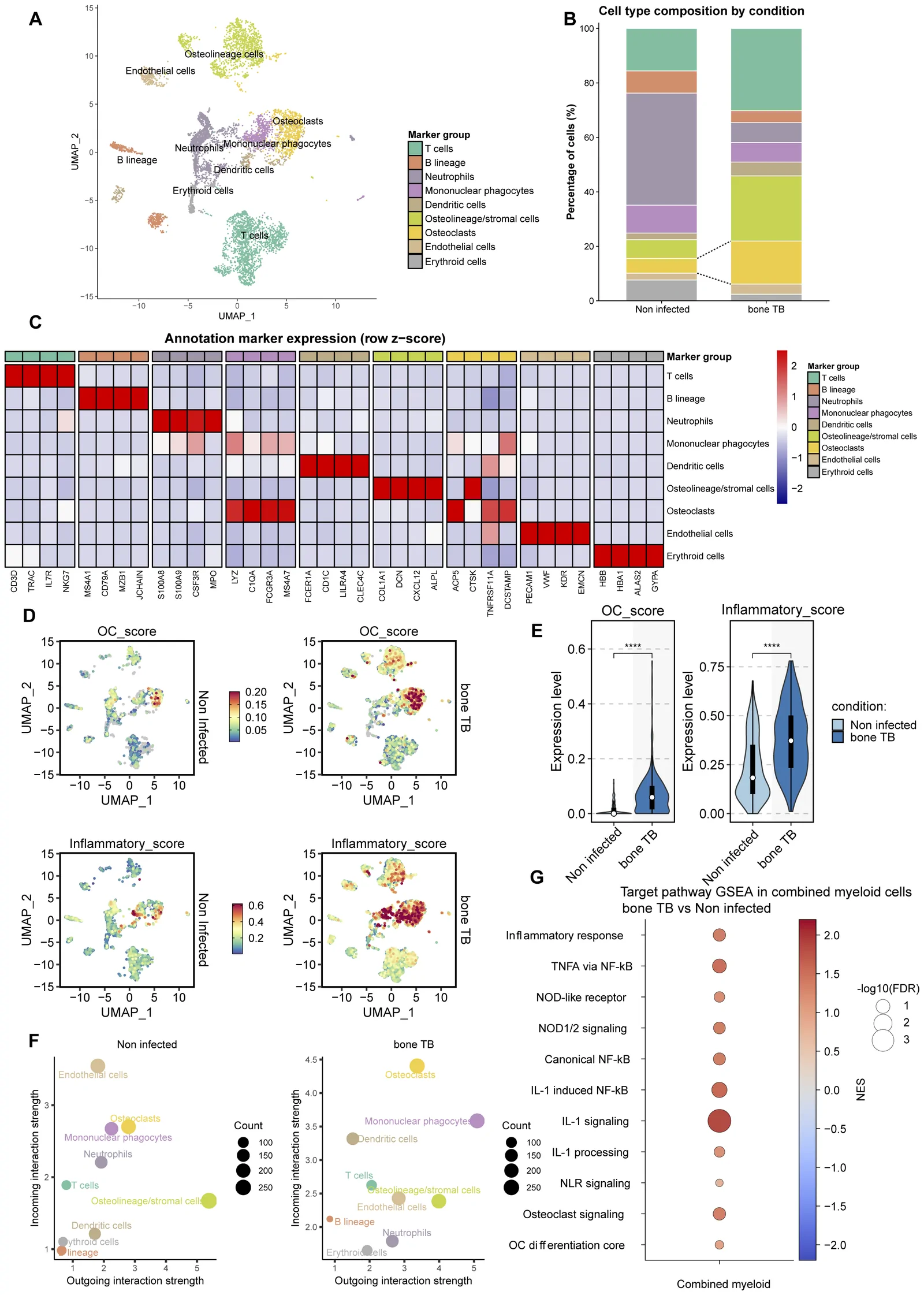
Human Single-Cell Analysis Reveals Coordinated Inflammatory and Osteoclastogenic Remodeling in Bone Tuberculosis. **(A)** UMAP of integrated single-cell RNA-sequencing data from human bone tuberculosis tissues and non-infected reference samples, showing nine major annotated cell populations. **(B)** Aggregate cell-type composition in non-infected and bone tuberculosis samples. **(C)** Heatmap of canonical marker expression used to support cell-type annotation. **(D)** Condition-split UMAPs showing per-cell osteoclast-differentiation and inflammatory UCell scores in non-infected and bone tuberculosis samples. **(E)** Violin plots showing the per-cell distributions of osteoclast-differentiation and inflammatory scores by condition. **(F)** CellChat-inferred global incoming and outgoing interaction strengths for the major cell populations in non-infected and bone tuberculosis samples. **(G)** Targeted gene-set enrichment analysis of combined myeloid cells comparing bone tuberculosis with non-infected samples.

Cell–cell communication analysis inferred a disease-associated shift. In non-infected samples, endothelial cells received the most incoming signals and osteolineage/stromal cells sent the most outgoing signals. In TB, mononuclear phagocytes became the dominant outgoing source, while osteoclasts emerged as the primary incoming target (**Figure 4F**). This pattern is consistent with a model in which activated myeloid cells drive signaling toward osteoclast-lineage cells.

Targeted gene-set enrichment on combined myeloid cells showed positive TB-associated enrichment for inflammatory response, TNF–NF-κB, NOD-like receptor, NOD1/2, canonical NF-κB, IL-1 signaling, inflammasome, and osteoclast pathways (**Figure 4G**). Altogether, these results define a TB myeloid environment marked by inflammatory activation, altered cell-cell communication, and osteoclastogenic priming signaling.

### 3.5 RIPK2 expression is associated with inflammatory osteoclastogenic states in human and experimental bone tuberculosis

We next turned to human bone TB tissues to examine RIPK2 expression in the disease context. Compared with non-infected references, RIPK2 was more broadly expressed in TB samples, especially within myeloid and osteoclast-associated compartments. IL1B co-localized in a similar pattern, consistent with an inflamed myeloid environment (**Figure 5A**).

**Figure 5:**
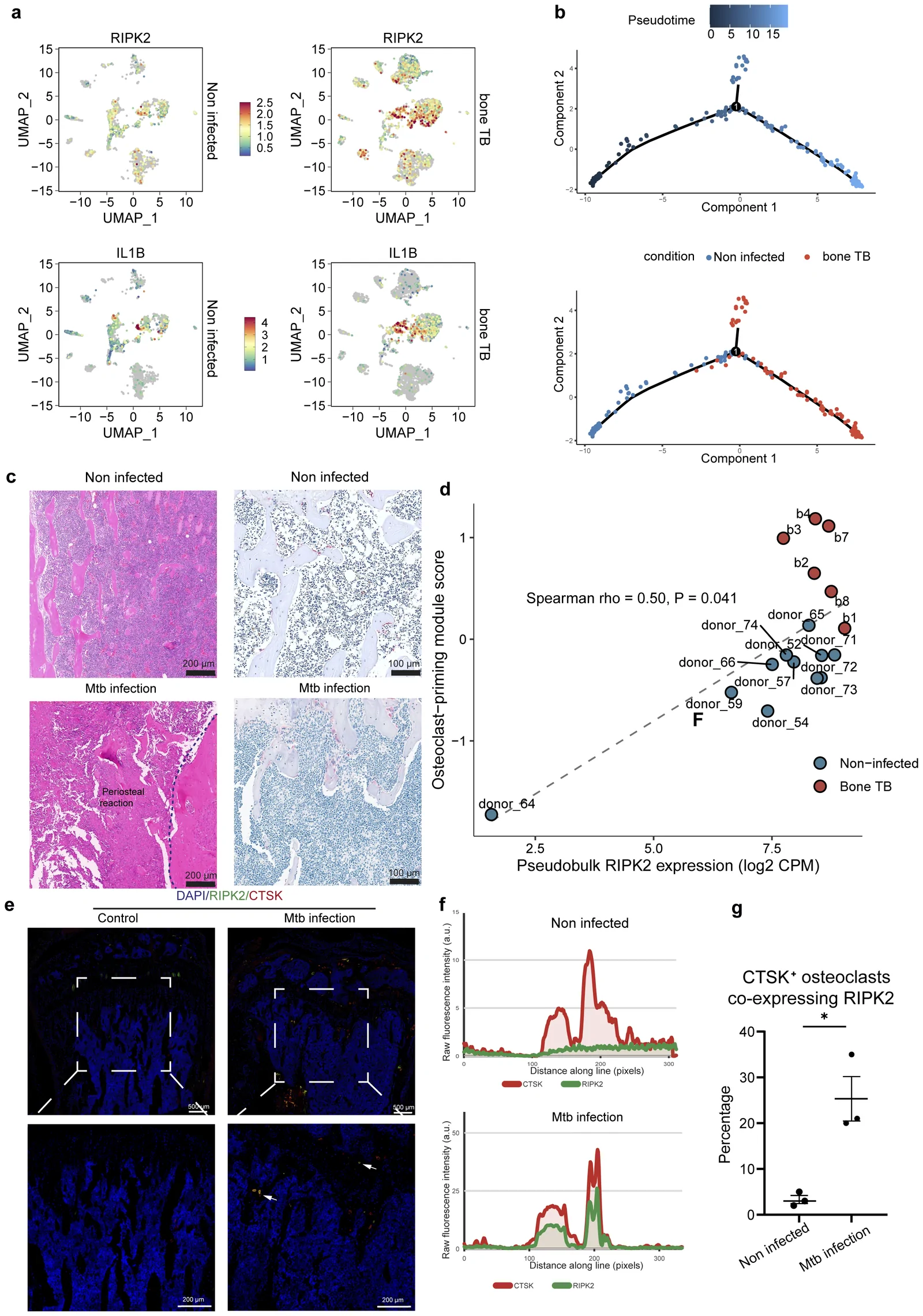
RIPK2 Expression Is Associated with Inflammatory Osteoclastogenic States in Human and Experimental Bone Tuberculosis. **(A)** Condition-split UMAPs showing normalized RIPK2 and IL1B expression in human non-infected reference and bone tuberculosis samples. **(B)** Monocle2 trajectory analysis of the mononuclear-phagocyte–osteoclast compartment. **(C)** Representative hematoxylin and eosin staining and tartrate-resistant acid phosphatase staining of non-infected and Mtb-infected mouse bone. Scale bars: 200 μm for hematoxylin and eosin staining and 100 μm for tartrate-resistant acid phosphatase staining. **(D)** Donor-level association between mononuclear-phagocyte pseudobulk RIPK2 expression and the osteoclast-priming module score across the combined non-infected reference and bone tuberculosis cohort. **(E)** Representative immunofluorescence staining of non-infected and Mtb-infected mouse bone sections. Nuclei are shown by DAPI in blue, RIPK2 in green, and CTSK in red. Scale bars in the upper panels: 500 μm, in the lower panels: 200 μm. **(F)** Representative line-scan fluorescence-intensity profiles across the regions indicated in panel **(E, G)** Quantification of the percentage of CTSK-positive osteoclast-lineage cells co-expressing RIPK2 in non-infected and Mtb-infected mouse bone, n=3. Data are presented as mean ± SEM.

Monocle2 on the mononuclear-phagocyte–osteoclast subset generated a trajectory that started from early myeloid states and ended at osteoclast-associated states. TB-derived cells extended further into the later pseudotime regions (**Figure 5B**). Pseudobulk RIPK2 expression in mononuclear phagocytes correlated positively with the osteoclast-priming module score at the donor level (Spearman’s ρ = 0.50, P = 0.041; **Figure 5D**). These human data pointed to a link between RIPK2 and inflammatory osteoclastogenic states.

We then checked whether this relationship held up *in vivo*. In a mouse TB model, H&E staining revealed inflammatory lesions with periosteal reaction, and TRAP staining highlighted osteoclast-lineage cells along infected bone surfaces (**Figure 5C**). Immunofluorescence showed that RIPK2 signal was elevated in CTSK-positive bone-interface regions of infected sections (**Figure 5E**). Both colocalization and quantitative analyses confirmed greater spatial overlap between RIPK2 and CTSK after infection, along with a higher proportion of RIPK2^+^CTSK^+^ osteoclast-lineage cells (**Figures 5F, G**). Taken together, the human and mouse data place RIPK2 at osteoclast-rich lesion sites and link it to inflammatory osteoclastogenic progression, without implying that RIPK2 directly drives osteoclast differentiation.

### 3.6 GSK583 attenuates Mtb lysate-induced inflammatory priming and inhibits RANKL-driven osteoclastogenesis

We next examined bone microarchitecture in the mouse TB model. MicroCT revealed disrupted trabecular architecture in Mtb-infected bone, which showed wider trabecular separation and fewer trabeculae than in uninfected controls (**Figures 6A, B**).

**Figure 6:**
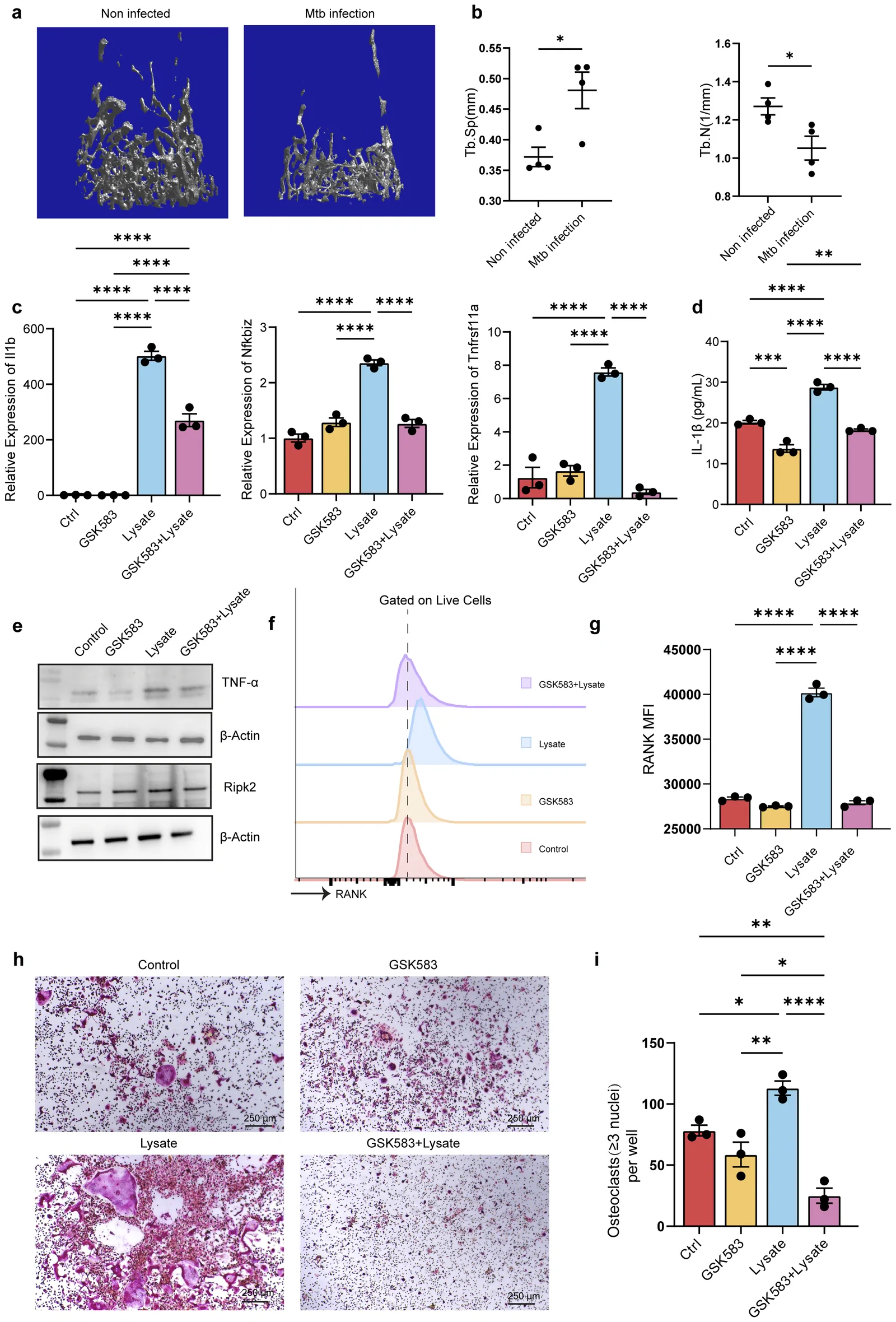
GSK583 attenuates Mtb lysate-induced inflammatory priming and inhibits RANKL-driven osteoclastogenesis. **(A)** Representative microcomputed-tomography reconstructions of trabecular bone from non-infected and Mtb-infected mice. **(B)** Quantification of trabecular separation (Tb.Sp) and trabecular number (Tb.N). **(C)** RT-qPCR analysis of *Il1b*, *Nfkbiz*, and *Tnfrsf11a* expression in primary BMDMs treated for 24 hours with vehicle control, GSK583 (1 μM), heat-killed Mtb lysate (10 μg/mL), or GSK583 plus Mtb lysate. **(D)** IL-1β concentrations in culture supernatants measured by ELISA after 24 hours of treatment; *n* = 3 independent biological experiments per group. **(E)** Representative immunoblots showing TNF-α and RIPK2 protein abundance, with β-actin as the loading control. **(F)** Representative flow-cytometric distributions of cell-surface RANK in live BMDMs after 24 hours of treatment. **(G)** Quantification of RANK median fluorescence intensity; *n* = 3 independent biological experiments per group. **(H)** Representative tartrate-resistant acid phosphatase staining of BMDMs differentiated with M-CSF and RANKL in the presence or absence of Mtb lysate and GSK583. Scale bars: 250 μm. **(I)** Quantification of tartrate-resistant acid phosphatase-positive multinucleated osteoclasts containing at least three nuclei. *n* = 3 independent biological experiments per group. Significance levels are indicated as *p < 0.05, **p < 0.01, and ***p < 0.001, ****P < 0.0001.

To explore the underlying cellular response, we used heat-killed Mtb lysate as a standardized stimulus in primary BMDMs and asked whether GSK583 could modulate the effects. Dose–response CCK-8 assays ruled out significant metabolic effects at 0.5–2 μM GSK583 or 10–50 μg/mL lysate over 24 hours, so we proceeded with 1 μM and 10 μg/mL (**Supplementary Figure S1B**). Lysate treatment increased Il1b, Nfkbiz, and Tnfrsf11a expression, elevated IL-1β secretion, and raised TNF-α signals on immunoblots; GSK583 co-treatment reduced each of these responses (**Figures 6C–E**). Surface RANK fluorescence followed the same pattern—higher with lysate, lower when GSK583 was added (**Figures 6F, G**). Functionally, under RANKL-driven differentiation conditions, Mtb lysate increased TRAP-positive multinucleated osteoclast formation, consistent with enhanced osteoclastogenic responsiveness, whereas GSK583 attenuated this lysate-associated effect (**Figures 6H, I**). Overall, Mtb lysate induces a GSK583-sensitive inflammatory and RANK-priming program, and GSK583 blocks the lysate-potentiated, RANKL-driven osteoclastogenesis.

## 4 Discussion

Macrophages and osteoclasts share a common myeloid lineage, and our data suggest that Mtb-derived inflammatory stimulation primes macrophages for enhanced responsiveness to RANKL rather than directly inducing osteoclast differentiation. The dual upregulation of pro-inflammatory cytokines (*Il1b*, IL-1β) and RANK-associated genes (*Tnfrsf11a*, surface RANK) positions macrophages as a cellular bridge between bacterial sensing and bone resorption. This is consistent with the classical model that inflammatory cytokines lower the threshold for RANKL-driven osteoclastogenesis (Zhou et al., 2022; Rauner and Bozec, 2025). Single-cell profiling of human bone tuberculosis reinforced this connection: myeloid populations showed broader inflammatory and osteoclast-differentiation programs and occupied later stages of a monocyte-to-osteoclast trajectory, underscoring a disease-relevant inflammatory–osteoclastogenic program.

RIPK2 emerged from our screen as a node supported by super-enhancer activation, transcriptional concordance, and chromatin-contact context. Upon mycobacterial infection, muropeptides such as muramyl dipeptide (MDP) exit the phagosome via SLC15A family transporters and are sensed by NOD2. NOD2 then recruits RIPK2 through CARD-CARD domain interactions; RIPK2 is phosphorylated and undergoes Lys63-linked polyubiquitination, which mediates downstream signaling cascades culminating in NF-κB and MAPK activation (Strober and Watanabe, 2011; Honjo et al., 2021). Notably, RIPK2 polyubiquitination is stimulated when macrophages are infected with live Mycobacterium tuberculosis (Dube and Behr, 2023). The NOD2-RIPK2 axis has been directly implicated in macrophage responses to M. tuberculosis, including inflammatory and type I interferon programs (Pandey et al., 2009). Genetic evidence further supports the relevance of RIPK2 in tuberculosis susceptibility: a recent study in a Western Chinese Han population identified that the G allele of the RIPK2 rs39509 polymorphism is associated with increased TB risk (Song et al., 2019).

The pharmacological suppression of both inflammatory and osteoclastogenic readouts by GSK583 reinforces the notion that RIPK2 activity is required for the full expression of the lysate-induced program. GSK583 is an ATP-competitive, type I kinase inhibitor that specifically targets the kinase domain of RIPK2 (Haile et al., 2016). While RIPK2 inhibitors have been primarily evaluated in the context of inflammatory bowel disease and other sterile inflammatory conditions (Tigno-Aranjuez et al., 2014; Canning et al., 2015; Lai et al., 2023), our data extend this pharmacology into infectious bone pathology. An interesting implication is that the same NOD2-RIPK2 module may operate differently depending on the tissue context: in the gut, it drives mucosal inflammation; in bone, it may also license osteoclast precursors to respond to RANKL. Importantly, the fact that GSK583 attenuated RANK priming without directly blocking RANKL signaling suggests that the inhibitor acts upstream of the RANK receptor, which may offer an advantage over direct anti-RANKL approaches by also limiting the inflammatory milieu that drives osteoclastogenesis. GSK583 and related compounds illustrate this tractability through potent and selective RIPK2 inhibition and suppression of muramyl dipeptide-induced cytokine responses in cellular systems (Haile et al., 2016). Our findings extend this pharmacological rationale to bone tuberculosis by showing that GSK583 modulates macrophage inflammatory activation, RANK-associated priming, and osteoclastogenic output. This possibility is relevant because current treatment of bone tuberculosis is centered on prolonged antimicrobial therapy and, in selected cases, surgery, whereas host-directed strategies aimed at limiting inflammation-associated bone resorption remain underdeveloped (Leonard and Blumberg, 2017; Deng et al., 2018).

Several limitations should be noted. The super-enhancer–gene assignments and Hi-C analyses remain predictive rather than functional validations of enhancer activity. Heat-killed Mtb lysate standardizes mycobacterial stimulation but cannot recapitulate replication-dependent or viability-dependent host–pathogen interactions. Our TB samples were generated in-house; controls came from public datasets. Harmony integration cannot fully eliminate residual technical or cohort differences. We therefore treat cross-condition comparisons as disease-associated patterns and used donor-level summarization for pseudobulk and correlation analyses. These results therefore provide pharmacological proof of concept that warrants future *in vivo* intervention studies.

In summary, the integrated multi-omic and experimental data define a RIPK2-associated macrophage inflammatory program that couples RANK priming to osteoclastogenic output in bone tuberculosis. These findings support further evaluation of RIPK2-directed intervention as a host-directed strategy to limit inflammatory bone destruction.

## Data Availability

The datasets presented in this study can be found in online repositories. The names of the repository/repositories and accession number(s) can be found in the article/**Supplementary Material**.
